# Barriers and Facilitators of Medicaid Participation Among Dentists

**DOI:** 10.1001/jamahealthforum.2025.4403

**Published:** 2025-11-21

**Authors:** Hawazin W. Elani, Niran Prakash, Renuka Tipirneni

**Affiliations:** 1 Department of Oral Health Policy and Epidemiology, Harvard School of Dental Medicine, Harvard University, Boston, Massachusetts; 2 Department of Health Policy and Management, Harvard T.H. Chan School of Public Health, Harvard University, Boston, Massachusetts; 3 Health and Human Services Commission, Austin, Texas; 4 Division of General Medicine, Department of Internal Medicine, University of Michigan, Ann Arbor; 5 Institute for Healthcare Policy and Innovation, University of Michigan, Ann Arbor; 6 Department of Health Management and Policy, School of Public Health, University of Michigan, Ann Arbor; 7 Division of Hospital Medicine, Department of Internal Medicine, University of Michigan, Ann Arbor

## Abstract

**Importance:**

Dentists’ limited participation in Medicaid is a substantial barrier to addressing the persistent unmet need for dental care among adult Medicaid beneficiaries.

**Objective:**

To assess dentists’ experiences and perceptions regarding Medicaid and to identify barriers and facilitators to participation as well as strategies to improve dentists’ participation and service delivery.

**Design, Setting, and Participants:**

This qualitative study involved semistructured individual interviews of dentists from 8 states (Maryland, Massachusetts, Michigan, New York, North Carolina, South Dakota, Wisconsin, Wyoming) about their experiences with and perceptions of Medicaid. The study was conducted between August 2022 and July 2023. Data were analyzed from September 2023 to September 2024.

**Main Outcomes and Measures:**

Themes related to Medicaid participation were identified through thematic analysis, and descriptive statistics were used to characterize the sample.

**Results:**

Of the 67 dentists interviewed, 46 (68.6%) accepted Medicaid, and 21 (31.3%) did not accept Medicaid. The sample consisted of 36 females (53.7%) and 31 males (46.3%), with a mean (SD) age of 45.1 (14.4) years and a mean (SD) of 16.4 (13.8) years of work experience. Three key domains affecting dentists’ participation in Medicaid were identified: system-level, dentist-level, and patient-level factors. At the system-level domain, barriers to Medicaid acceptance, including low reimbursement rates, administrative burdens, restrictive benefit designs, and poor communication about benefits, played a role in perceived inefficiencies and limited dentists’ engagement. Challenges at the dentist-level domain included language barriers, capacity constraints, stigma surrounding Medicaid, and concerns about financial sustainability. Factors at the patient level, including appointment adherence, unfavorable perceptions of preventive care, logistical barriers, and limited oral health literacy, had a potential role in limiting effective care delivery. Dentists who accepted Medicaid reported frustration with administrative inefficiencies and reimbursement rates, whereas those who did not participate in Medicaid emphasized financial stability concerns.

**Conclusions and Relevance:**

This study highlights the complex interplay of barriers and facilitators at system, dentist, and patient levels that impact oral health care delivery to adult Medicaid beneficiaries. Increasing reimbursement rates is important but should be complemented by efforts to streamline administrative processes, improve patient engagement, and support dentists through targeted incentive programs.

## Key Points

**Question:**

What are dentists’ experiences and perceptions regarding Medicaid participation, and what barriers and facilitators affect their decision to accept or decline Medicaid insurance?

**Findings:**

In this qualitative study of 67 dentists across 8 states, themes related to Medicaid participation were identified and organized into system-, dentist-, and patient-level factors. Dentists consistently reported low reimbursement, administrative burden, and no-shows as barriers to participation.

**Meaning:**

Findings of this study suggest that improving Medicaid dental access requires policy efforts beyond reimbursement increases, including reducing administrative burdens and supporting dentist engagement.

## Teaser

This qualitative study describes dentists’ perceptions of Medicaid participation and the barriers and facilitators that play a role in their decision to participate in the program.

## Introduction

The provision of dental care to adult Medicaid beneficiaries has long been challenged by systemic inefficiencies, financial constraints, and disparities in access.^1^ Despite Medicaid’s key role as a safety net for populations with low income, adult dental benefits are often limited and fall short of addressing comprehensive oral health needs.^2,3^ While Medicaid mandates dental coverage for children, coverage for adults is frequently limited, resulting in states’ administration of their programs. As of 2024, 9 states did not cover any dental benefits, 8 offered limited coverage, and 34 offered comprehensive benefits (beyond preventive visits and simple fillings), often with annual caps or service limits.^4^ However, in states with comprehensive coverage, mean use of any dental service remains low at 34%, with lower rates in rural areas due to dentist shortages.^5,6^

Dentists’ limited participation in Medicaid is a substantial barrier to the program’s effectiveness in improving access to oral health care for adults with low income.^5,7^ Nationally, only 43% of dentists participate in Medicaid,^8^ with even lower rates in some states. For example, more than half of dentists in North Carolina and nearly three-quarters in Virginia do not participate in Medicaid.^9,10^ This reluctance is often attributed to inadequate reimbursement rates and administrative burdens.^5^ These access barriers are especially concerning in rural and underserved areas, where many Medicaid beneficiaries already face limited access to dental care.^6^

Expanding access to dental care through Medicaid depends on dentists’ engagement. To address barriers that limit their participation, it is important to understand dentists’ perceptions of Medicaid and the factors that affect their decision to participate. This qualitative study explores dentists’ experiences with Medicaid, including barriers and facilitators to participation, to provide insights into the complexities of Medicaid dental care access and identify strategies to improve dentists’ participation and service delivery.

## Methods

The study was approved by the Institutional Review Board of the Harvard Faculty of Medicine. All participants provided verbal and written informed consent, which included permission for audio recording and use of deidentified quotes for research purposes. We followed the Consolidated Criteria for Reporting Qualitative Research (COREQ) reporting guideline.^11^

### Recruitment

We used a stratified purposeful sampling strategy to select 8 states based on their Affordable Care Act Medicaid expansion status and Medicaid reimbursement rates.^12^ Our sampling frame included 34 states offering adult Medicaid dental benefits beyond emergency coverage. From this pool, we selected 4 expansion and 4 nonexpansion states, ensuring variation in Medicaid reimbursement rates. Reimbursement rates were defined as a percentage of private dental insurance reimbursement based on 2020 data.^13^ The selected states (Maryland, Massachusetts, Michigan, New York, North Carolina, South Dakota, Wisconsin, and Wyoming) also had relatively similar demographic characteristics.^14^

To recruit participants, we collaborated with professional organizations, including state dental associations, health center directors, state dental directors, and the American Dental Education Association. These groups distributed study information through online forums, social media, and email. Interested dentists contacted the research team and were screened for eligibility. We also used snowball sampling, encouraging participants to refer to other eligible dentists. Eligible participants were licensed dentists practicing in the selected states who spoke English and treated adult patients.

### Interviews

Guided by the Social Ecological Model^15^ and previous research on dental care access barriers,^16,17,18,19,20^ we conducted semistructured interviews to explore dentists’ perspectives and experiences regarding Medicaid participation. The interview guide included open-ended questions on Medicaid participation history, program perceptions, attitudes toward beneficiaries, and suggestions for program improvement (eMethods in Supplement 1). Prior to interviews, participants completed a brief survey, providing information on age, sex, race, ethnicity, years of experience, clinician type, practice setting, state, and location. Race and ethnicity were collected to characterize the sample and not analyzed further. Participants self-reported race and ethnicity including Asian, Black, or White race and Hispanic or non-Hispanic ethnicity. We piloted the guide with 2 dentists who did not participate in the primary study and refined the questions based on feedback.

Each interview began with questions about Medicaid participation. For dentists who accepted Medicaid, we explored their experiences with reimbursement rates, administrative processes, and interactions with Medicaid beneficiaries, as well as the implications of Medicaid for their practice and comparisons with privately insured or self-paying patients. Dentists who did not accept Medicaid were asked about their reasons for nonparticipation and their views on the program’s implications for access to dental care. All interviews concluded with questions about recommendations to improve Medicaid dental services and an opportunity to share any additional insights.

Interviews were conducted by a trained qualitative researcher via videoconferencing software (Zoom Communications Inc), lasted approximately 1 hour, and were audio-recorded for transcription. Participants received a $50 Amazon gift card. Interviews took place from August 2022 to July 2023 until thematic saturation was reached.^21^

### Data Analysis

We conducted a verbatim transcription of all interviews and used thematic analysis.^22^ Two researchers (H.W.E. and N.P.) independently coded a subset of interviews to develop an initial codebook, which they refined through regular meetings. We used an iterative process to group codes, identify and conceptually map themes, and organize findings using matrices. We conducted all data management and analysis from September 2023 to September 2024 using NVivo 14 (Lumivero).

## Results

We conducted interviews with 67 dentists across 8 states (Table 1). Among the participants, 46 accepted Medicaid, and 21 did not. Of these participants, 31 (46.3%) were male and 36 (53.7%) were female, with a mean (SD) age of 45.1 (14.4) years and a mean (SD) of 16.4 (13.8) years in practice. Most participants were general dentists (46 [68.7%]). Overall, dentists in the sample practiced in academic centers (12 [17.9%]), community health centers (20 [29.9%]), or private practice (35 [52.2%]). Compared with national data, our sample included more female, younger, and Medicaid-participating dentists.^23^

**Table 1.** Sociodemographic Characteristics of the Study Participants

Characteristic	Participants, No. (%)^a^		
	Full sample (N = 67)	Accepts Medicaid (n = 46)	Does not accept Medicaid (n = 21)
Age, mean (SD), y	45.1 (14.4)	44.4 (14.1)	55.7 (15.9)
Sex			
Female	36 (53.7)	26 (56.5)	11 (52.3)
Male	31 (46.3)	20 (43.4)	10 (47.6)
Race			
Asian	15 (22.4)	10 (21.7)	5 (23.8)
Black	7 (10.4)	5 (10.9)	2 (9.5)
White	45 (67.2)	31 (67.4)	14 (66.7)
Ethnicity			
Hispanic	9 (13.4)	4 (8.7)	5 (23.8)
Non-Hispanic	58 (86.6)	42 (91.3)	16 (76.2)
Years of experience, mean (SD)	16.4 (13.8)	15.2 (12.7)	17.6 (15.7)
Annual income, mean (SD), $ (thousands)	200.3 (64.6)	186.3 (56.3)	227.2 (71.5)
Clinician type			
General dentist	46 (68.7)	34 (73.9)	12 (60.0)
Specialist	21 (31.3)	12 (26.1)	9 (40.0)
Practice setting			
Private practice	35 (52.2)	20 (43.5)	15 (71.4)
Community health center	20 (29.9)	18 (39.1)	2 (9.5)
Academic center	12 (17.9)	8 (17.4)	4 (19.0)
Accepting new patients	61 (91.0)	40 (87.0)	21 (100)
Practice ownership	22 (32.8)	14 (30.4)	8 (38.0)
Location			
Rural	13 (21.0)	9 (20.0)	4 (19.0)
Urban	53 (79.0)	36 (80.0)	17 (81.0)
State^b^			
Maryland	12 (17.9)	9 (19.6)	3 (14.3)
Massachusetts	16 (23.9)	11 (23.9)	5 (23.8)
Michigan	12 (17.9)	8 (17.4)	4 (19.0)
New York	10 (14.9)	4 (8.7)	6 (28.6)
North Carolina	5 (7.5)	4 (8.7)	1 (4.8)
South Dakota	5 (7.5)	5 (10.9)	0
Wisconsin	5 (7.5)	4 (8.7)	1 (4.8)
Wyoming	2 (3.0)	1 (2.2)	1 (4.8)

^a^ Percentages may not sum to 100 due to rounding.

^b^ As of August 2022, Maryland, Massachusetts, Michigan, and New York had expanded Medicaid under the Affordable Care Act; North Carolina, South Dakota, Wisconsin, and Wyoming had not. Adult dental reimbursement rates expressed as a percentage of private dental insurance rates in 2020 were: 77.2% in Maryland, 50.8% in Massachusetts, 31.9% in Michigan, 71.0% in New York, 57.5% in North Carolina, 63.3% in South Dakota, 33.0% in Wisconsin, and 66.8% in Wyoming.^13^

The final analysis focused on system-level, dentist-level, and patient-level factors that play a role in Medicaid dental care delivery and dentists’ participation in the Medicaid program as well as suggestions for improving the program. Within each of these 3 domains, we identified key themes categorized as barriers or facilitators to Medicaid participation (Table 2).

**Table 2.** Domains and Themes Related to Dentists’ Perceptions of Medicaid Participation and Experiences

Domain and Theme	Description of barriers and facilitators of participation
**System-level factor**	
Funding and reimbursement	Barrier: low Medicaid reimbursement rates often fail to cover overhead costs, making participation financially unsustainable and discouraging dentists.
	Facilitator: state-affiliated health centers provide financial stability for dentists who accept Medicaid.
Administrative processes	Barriers: claim denials and administrative burdens create financial strain and inefficiencies. Credentialing and billing procedures vary across states, with some states having complex, lengthy processes.
	Facilitator: credentialing and billing procedures is streamlined in few states.
Prior authorization	Barrier: substantial delays in treatment approval lead to worsened dental conditions for patients.
Benefit design and coverage limitations	Barriers: insufficient coverage for essential services (eg, periodontal treatment) limits ideal treatment plans and compromises effective care. The financial challenge of balancing patient needs with limited coverage results in suboptimal care.
Communication about benefits	Barrier: the burden of educating patients about Medicaid program limitations often falls on dentists rather than the Medicaid system itself.
**Dentist-level factor**	
Capacity constraints	Barriers: high patient volume and operational inefficiencies pressure dentists to see more patients, forcing them to prioritize quantity over quality in care.
Stigma toward Medicaid	Barriers: unfavorable perceptions and biases surrounding Medicaid create stereotypes about patient appointment adherence and expectations, potentially playing a role in discrimination against beneficiaries.
Dentists’ motivation	Facilitators: many dentists remain committed to serving underserved populations because of a strong sense of duty and personal satisfaction. Incentives, such as loan repayment programs, may help sustain dentist participation.
**Patient-level factor**	
Appointment adherence and perceptions toward Medicaid	Barrier: misconceptions about publicly funded care play a role in a lack of patient accountability, leading to repeated last-minute cancellations and no-shows, wasting dentists’ time and revenue.
Attitude toward preventive care	Barrier: some patients prioritize immediate pain relief over preventive care, only seeking treatment when issues become severe rather than maintaining routine dental visits.
Structural and socioeconomic and barriers to care	Barriers: unpredictable work schedules, lack of paid sick leave, and multiple jobs make it difficult for patients to schedule and maintain dental appointments, especially during standard office hours.
Cultural and language barriers	Facilitator: the use of interpreters helps bridge communication gaps and may improve dentist-patient interactions.
Oral health literacy and affordability	Barriers: many patients are unaware of the importance of routine dental care, and Medicaid’s limited coverage often leads to substantial untreated oral health issues. Lack of routine access to dental care may lead to complex treatment needs that exceed coverage limits. The gap between treatment needs and Medicaid coverage can have substantial out-of-pocket costs, potentially leading some patients to disengage from care, miss appointments, or forgo follow-up treatments.

### System-Level Factors

Dentists consistently highlighted that Medicaid reimbursement rates were insufficient to cover overhead costs, describing them as “too low to make it sustainable.” Dentists also emphasized the gap between Medicaid and private insurance rates. The tension between financial sustainability and patient care was a recurring theme: “We have to be part of the solution. We are the providers. But at the same time, we have expenses. For a provider to stay afloat, we need to be 25% profitable, and then 75% is overhead, and so, for every dollar, if we’re only paid 33%, where are you going to get the rest of the money to pay for your assistant, for your place, for your supplies? The formula has to work.” This dentist was referring to the financial reality of private practice, where at least a 25% profit margin is needed to remain sustainable after covering overhead costs.

Participants reported that the high costs of operating a dental practice and student debt, which often exceed $300 000, added to financial strain: “The front-loading of costs to become a dentist and run a business is significant.” Student loans also limited dentists’ participation: “We have to pay back our student loans. So it [seeing Medicaid beneficiaries] becomes a barrier for us because we can’t really afford it. And we have to pay back so much debt.”

These financial pressures add to a cycle of financial losses, discouraging Medicaid participation. Dentists said reimbursements were disproportionately low compared with private insurance rates, making it difficult to break even: “If they’re paying $50 or $100 for an extraction, and I’m spending 45 minutes, it just doesn’t make sense” and “if I’m doing a crown, after materials, after paying staff and lab fees, if they’re only paying $400. Then how much is the dentist actually making?”

Dentists reported that limited and inconsistent Medicaid coverage restricted essential care, leading to gaps in treatment, with 1 dentist stating that “They [Medicaid] would cover a crown, but they wouldn’t cover the procedure in between. You can’t go from a root canal to not having a core build-up to having a crown, so you just have to do that procedure for nothing.” These gaps often forced dentists to either absorb the costs themselves or deny care they considered necessary.

Dentists also expressed frustration when discussing treatment options with patients unaware of Medicaid’s coverage limitations. “Imagine how frustrating it is to hear like, you could get a bridge, but you have to pay out of pocket $2400, and if you just go with this other option, to have to pull your teeth, I know that’s embarrassing for you, but that’s really the only option insurance will cover. The patients get really frustrated. Medicaid favors pulling teeth, so it gets really frustrating.”

Administrative inefficiencies such as slow, unpredictable Medicaid claims, reimbursement delays, and prior authorization taking up to 2 months added burden to providing dental care to Medicaid beneficiaries. As one dentist described, “You submit a claim, and they can’t get a predetermination or a preauthorization back in time, it’s definitely frustrating.” However, some reported a smoother enrollment process (credentialing) and more responsive agency support staff, particularly in Massachusetts and New York, both Medicaid expansion states with relatively higher dental reimbursement rates, suggesting that administrative infrastructure may vary substantially across states and could offer models for improvement. Dentists in state-affiliated health centers also described more financial stability, enabling them to participate with fewer financial losses.

### Dentist-Level Factors

Dentists cited heavy workloads and overburdened practices as major challenges to Medicaid participation. Due to low reimbursement, many dentists felt compelled to see more patients in less time to maintain financial viability: “The volume is increased to compensate for the low fees.” This pressure prevented dentists from providing the level of care they desired: “You can’t give the person the time that they deserve.” Consequently, many dentists reported finding themselves prioritizing quantity over quality, leading to burnout and dissatisfaction.

Some dentists discussed the stigma associated with Medicaid. Unfavorable perceptions often led to assumptions about patient behavior, particularly regarding appointment adherence and expectations. These biases contributed to systemic discrimination against Medicaid beneficiaries, with one dentist stating that “Racism and bias play a role as well, in terms of people’s perceptions of the Medicaid population.”

Among dentists who accepted Medicaid, many remained committed to serving Medicaid patients despite ongoing challenges. Their motivation was driven by a strong sense of social responsibility: “It’s my social duty and ethical obligation. As dentists, we took an oath to take care of people. So, I think that if everyone does a little bit, we can go a long way.” For many dentists interviewed, the desire to give back and help people was a reason for their participation in Medicaid. Some dentists also viewed their participation as playing a role in broader systemic change, stating they wanted to be “part of the solution” in improving access to dental care.

### Patient-Level Factors

Many dentists reported high no-show rates for patients with Medicaid, which disrupted their schedules and caused financial losses. “The big problem is the no-show rate, and you can’t bill them for a missed visit. The no-show rate can be very high, which is a wasted appointment time.” Some dentists tried to compensate by double-booking appointments, which led to burnout and overwhelming workloads. “I’m triple-booked at some points. It’s one of those things that gets frustrating. Just this morning, I walked in, and I had an extraction, an emergency exam, and a comprehensive exam all first thing in the morning…and then I still have 15 more patients on my schedule.”

Some dentists attributed no-shows to a perceived lack of accountability and misconceptions about “free” Medicaid coverage: “I think the free factor is definitely part of it. If they’re not committed, then why do I need to show up unless it hurts?” Dentists also felt many patients only sought care when experiencing pain rather than attending preventive visits: “They don’t show up unless there’s an emergent need, they’re hurting, and they have other priorities.”

Limited oral health literacy was also perceived to affect patient engagement. Dentists felt many Medicaid beneficiaries lacked awareness of the importance of routine dental care and coverage limits, leading to a focus on immediate relief over preventive care: “There is a certain population that is always an emergency only, and they only show up when they have a problem. They don’t want to follow through with things. They definitely do not want the treatment plan presented to them where items are not covered under their insurance.” Gaps between patients’ needs and Medicaid coverage frequently resulted in high out-of-pocket costs, causing some Medicaid enrollees to forgo care altogether: “Insurance may drive the treatment plan. If insurance covers extraction and doesn’t cover a crown, we present all treatments and we recommend the patient save the tooth, but the patient may decide, ‘I don’t want to pay.’” As a result, extractions were frequently prioritized over restorative treatments.

Beyond behavioral factors, dentists reported structural and socioeconomic barriers contributing to missed appointments. Many Medicaid beneficiaries had unpredictable work schedules and limited paid sick leave: “They don’t have sick time necessarily. [With] their work schedules, they wouldn’t know until sometimes the day before or the day of [if they could attend the appointment].” Lack of transportation is another barrier for Medicaid beneficiaries that may play a role in missed appointments: “They have a lot of social issues going on in their lives, so it’s just like a lot of factors that can make it challenging.”

Language barriers and cultural misunderstandings were also reported: “It is a communication issue. A lot of the Medicaid patients don’t necessarily speak English, so the translator involved slows things down and makes it more difficult to communicate.” However, dentists in community health settings reported that interpreters and cultural competency training improved communication and patient care. “Community health workers make a huge difference. They provide context for patients’ needs and that really helps.”

### Suggestions for Promoting Dental Medicaid Access

Dentists proposed several reforms to improve Medicaid participation (Table 3). Key recommendations included increasing reimbursement rates to make participation financially sustainable, expanding coverage to services like crowns and periodontal therapy, and offering financial incentives such as loan repayment and tax credits.

**Table 3.** Participants’ Suggestions for Promoting Dentists’ Participation in Medicaid

Domain and recommendation	Quote
**System-level factor**	
Increase reimbursement rates	“Money talks, so if [dentists] are not getting reimbursed anywhere near what private insurers will reimburse, then there’s no incentive to take patients” —Participant who accepts Medicaid, works at a community health center, and has 6-15 y in practice
Expanding covered services	“If Medicaid is going to cover root canal on molar tooth, then it should cover the crown as well, but that’s not the case. It should have a package that’s really tightly knit so that it represents what should be provided for.” —Participant who does not accept Medicaid, works in private practice, and has ≥20 y in practice
Enhance incentives for Medicaid participation	“If 35% of your patient base is Medicaid patients, then you get some type of tuition reimbursement or loan repayment incentive. That’s the language that will get our ears because we’re graduating with so much debt.” —Participant who does not accept Medicaid, works in private practice, and has 0-5 y in practice
	“Where the money you make from [treating Medicaid patients] is tax-free, that’s an incentive for somebody to take Medicaid patients. At least I know that after collecting the reimbursement, I know that it’s worth my time.” —Participant who does not accept Medicaid, works in private practice, and has 0-5 y in practice
Rebrand Medicaid program to reduce stigma	“They should definitely change the name, so like use private insurance names, use like a different name other than Medicaid because just by saying it’s a Medicaid program that automatically puts a lot of prejudice, and it's usually negative prejudices.” —Participant who does not accept Medicaid, works at an academic center, and has ≥20 y in practice
**Dentist-level factor**	
Strengthen dental education and training	“I do think it’s important to have a positive experience working with Medicaid patients at the training level. When students have a good experience, I think that would shape their attitude towards the program.” —Participant who accepts Medicaid, works at a community health center, and has 6-15 y in practice
**Patient-level factor**	
Enhance patient accountability and engagement	“I think people need to have some skin in the game. Even a small copayment on a sliding fee scale, however small it is, doesn’t matter. I think people appreciate more when they have to make a sacrifice.” —Participant who accepts Medicaid, works in a private practice, and has 6-15 y in practice

Several dentists also advocated for a shared responsibility model, proposing small patient copays to increase accountability and reduce no-show rates. Others recommended rebranding the Medicaid program to reduce stigma and emphasized the importance of dental education and outreach for future dentists and Medicaid beneficiaries.

## Discussion

This study highlights 3 key domains that have implications for dentists’ participation in Medicaid. System-level barriers, including administrative burdens, restrictive benefit designs, and inadequate funding, were perceived to play a role in inefficiencies and limited dentists’ engagement. Dentist-level challenges included stigma related to Medicaid and financial sustainability concerns. Dentists who accepted Medicaid reported frustration with administrative inefficiencies and reimbursement rates, while those who opted out emphasized financial viability concerns. Patient-level factors, including low appointment adherence, unfavorable attitudes toward preventive care, structural and cultural barriers, and limited oral health literacy, were also perceived by dentists to be factors that limited effective care delivery. Despite purposeful sampling across Medicaid expansion and nonexpansion states, dentists reported similar challenges and perspectives. These findings align with those reported by prior studies and emphasize dentists’ attitudes, perceived Medicaid program complexity, and motivation, regardless of policy structure or reimbursement level.^24,25^

Low reimbursement has long been a primary reason for dentists’ reluctance to participate in Medicaid, as many of them struggle to sustain their practices while treating high volumes of Medicaid patients. Although some states have increased dental reimbursement rates, changes in dentists’ participation have varied.^26,27^ In 2024, the mean Medicaid dental reimbursement was 49.8% of private insurance, with some states, such as New Hampshire, having a rate as low as 16.8%.^28^ While Medicaid rates are loosely tied to private insurance, the lack of a standardized approach creates wide variation. This study’s findings are consistent with prior studies; however, unlike most earlier studies focused primarily on dentists participating in Medicaid, this study includes perspectives from both participant and nonparticipant dentists across multiple states. While we did not specifically probe for experiences with Medicaid managed care organizations, prior research suggests variation in dentists’ engagement, and reimbursement may also influence dentists’ participation.^29^

Complex Medicaid credentialing, lengthy prior authorization, and inconsistent claims approvals added to the administrative burden for dentists and staff. While some states have improved these processes, efforts remain inconsistent. Modernizing Medicaid’s infrastructure through standardized electronic billing and faster claims processing could reduce these bureaucratic burdens. Additionally, leveraging artificial intelligence to handle preauthorizations and claims could improve efficiency, allowing dentists to prioritize patient care rather than navigating a complex bureaucracy.^30^

Coverage limitations also appeared to discourage dentist participation, often forcing dentists into a work-around mindset, requiring them to absorb costs themselves or omit necessary steps in standard care. For example, core build-ups may not be covered, despite being a necessary procedure between a covered root canal and a crown. Policymakers could consider establishing a federal minimum standard for Medicaid dental coverage to ensure more consistent care across states.

This study also describes how broader social determinants of health affect Medicaid beneficiaries. Dentists reported high rates of missed appointments, which disrupted scheduling and caused financial losses. Structural barriers, such as lack of transportation, unpredictable work schedules, and limited paid sick leave, prevent patients from consistently engaging with dental care services.^31^ While some Medicaid programs have begun to integrate transportation services, availability and quality vary. Scaling up these initiatives and incorporating more flexible scheduling could mitigate these barriers.^32^ Text reminders or small financial incentives for attendance could encourage patient engagement and minimize financial risk for dentists.^33^

Despite financial and administrative barriers, dentists’ motivation offers opportunities for policy innovation. Programs that recognize and reward dentists who participate in Medicaid, such as loan repayment, tax credits, or enhanced preventive care reimbursement, could help sustain participation. Expanding loan repayment eligibility beyond shortage areas to include dentists treating a threshold of Medicaid patients could boost engagement. States could partner with dental schools, expand teledentistry, and develop outreach programs to improve oral health literacy and access.^34,35,36^ Prior research indicates greater Medicaid participation among dentists practicing in rural settings, suggesting that practice context and workload may shape participation more than policy structure alone.^37^

### Limitations

The generalizability of our findings is limited by our sampling strategy. We purposefully selected 8 states based on their Medicaid expansion status and reimbursement rates, but the study does not capture the full diversity of Medicaid programs nationwide, particularly those with distinct local policies, workforce distribution, or extreme reimbursement variations. Additionally, our recruitment strategy may have introduced self-selection bias, as dentists with strong views on Medicaid, either favorable or unfavorable, may have been more likely to participate, potentially altering the themes that emerged.

## Conclusions

This qualitative study highlights the complex factors that play a role in dentists’ perceptions of Medicaid participation. Addressing these barriers requires coordination among state and federal policymakers, dental educators, and dentists. Increasing reimbursement rates is important but should be complemented by efforts to streamline administrative processes, improve patient engagement, and support dentists through targeted incentives.

## Notes

Supplement 1. **eMethods.** Interview Guide

Supplement 2.
Data Sharing Statement
