# Supplementary material for: Barriers and Facilitators of Medicaid Participation Among Dentists
Source: JAMA Health Forum. 2025 Nov 21;6(11):e254403. doi: 10.1001/jamahealthforum.2025.4403 (PMC12639478; doi:10.1001/jamahealthforum.2025.4403)
Supplement: Supplement 1. — eMethods. Interview Guide [file jamahealthforum-e254403-s001.pdf]

Supplemental Online Content

Elani HW, Prakash N, Tipirneni R. Barriers and Facilitators of Medicaid Participation Among Dentists. *JAMA Health Forum.* 2025;6(11):e254403. doi: 10.1001/jamahealthforum.2025.4403

eMethods. Interview guide

This supplemental material has been provided by the authors to give readers additional information about their work.

© 2025 Elani HW et al. *JAMA Health Forum*.

eMethods 1. Interview Guide

This semi-structured interview guide was developed to explore dentists' experiences, attitudes, and perceptions of Medicaid participation. Questions were guided by the Social Ecological Model and informed by prior literature on provider participation in public insurance programs. Interviews were conducted virtually via Zoom and lasted approximately 45–60 minutes.

==

Introduction script (read aloud by interviewer)

Thank you for agreeing to participate in this interview. We're interested in learning about your experiences with Medicaid and how this has influenced your practice and patients. There are no right or wrong answers. Everything you share will be de-identified in any reporting. This interview should take about 45 to 60 minutes. Do I have your permission to record this interview?

Section 1: Background and practice characteristics

1. Can you tell me a bit about your background in dentistry?
   - How long have you been practicing?
   - What is your specialty?
   - What kind of setting do you currently work in (e.g., private practice, FQHC, academic, other)?
2. What are the general demographics of the patient population you serve?
   - Would you describe your setting as urban or rural?

Section 2: Medicaid participation

3. Does your practice currently accept Medicaid or Medicaid managed care plans?
   - Approximately what percentage of your patients are Medicaid beneficiaries?
   - Are you currently accepting new Medicaid patients? Why or why not?
4. Have you ever accepted Medicaid in the past?
   - If so, why did you stop?
5. How has Medicaid participation (or non-participation) influenced your practice financially or operationally?

© 2025 Elani HW et al. *JAMA Health Forum*.

Section 3: Experience with the Medicaid system

6. Can you describe your experience with the administrative side of Medicaid? ○ Billing, credentialing, prior authorizations, claims processes?
7. How do you feel about the reimbursement rates for Medicaid services?
   - Do they cover your overhead? How do they compare to private insurance?
8. What has been your experience with the benefit structure of Medicaid?
   - Are there services or procedures not covered that impact your treatment planning?
9. Have you received any support or resources from state agencies or managed care organizations to assist with Medicaid navigation?

Section 4: Perspectives on Medicaid beneficiaries

10. How would you describe your experience treating patients who have Medicaid coverage?
    - Are there differences in expectations, adherence, or rapport compared to privately insured patients?
11. Have you observed any structural or social barriers that your Medicaid beneficiaries face in accessing care?
12. What are your perceptions of how Medicaid beneficiaries use preventive dental services?
13. How do you handle situations when recommended treatments are not covered under Medicaid?

Section 5: Values, motivation, and ethics

14. What motivates you to continue (or consider) participating in Medicaid?
15. Have you ever felt conflicted between providing the care your patient needs and what Medicaid will reimburse?
    - Can you describe an example?

Section 6: Recommendations and reflections

16. What changes would make Medicaid more feasible or appealing for dentists like you?
17. What could improve the experience of Medicaid beneficiaries in receiving dental care?
18. If you could speak directly to Medicaid policymakers, what would you want them to know?
19. Is there anything else you'd like to share about your experiences — either frustrations or stories of impact?

© 2025 Elani HW et al. *JAMA Health Forum*.

Closing script (read aloud by interviewer):

Thank you so much for your time and insights. Your input is incredibly valuable and will help inform efforts to improve dental care for Medicaid beneficiaries. Your responses will be anonymized in all reporting.

==
==

© 2025 Elani HW et al. *JAMA Health Forum*.
