# Supplementary material for: Barriers and Facilitators of Medicaid Participation Among Dentists
Source: JAMA Health Forum. 2025 Nov 21;6(11):e254403. doi: 10.1001/jamahealthforum.2025.4403 (PMC12639478; doi:10.1001/jamahealthforum.2025.4403)
Supplement: Supplement 2. — Data Sharing Statement [file jamahealthforum-e254403-s002.pdf]

Data Sharing Statement

Elani. Barriers and Facilitators of Medicaid Participation Among Dentists. *JAMA Health Forum*. Published November 21, 2025. doi:10.1001/jamahealthforum.2025.4403

Data
Data available: No
